# Selective serotonin re-uptake inhibitor sertraline inhibits bone healing in a calvarial defect model

**DOI:** 10.1038/s41368-018-0026-x

**Published:** 2018-09-03

**Authors:** R. Nicole Howie, Samuel Herberg, Emily Durham, Zachary Grey, Grace Bennfors, Mohammed Elsalanty, Amanda C. LaRue, William D. Hill, James J. Cray

**Affiliations:** 1 Oral Health Sciences, Medical University of South Carolina, Charleston, SC USA; 2 Biomedical Engineering, Case Western Reserve University, Cleveland, OH USA; 3 Cellular Biology and Anatomy, Augusta University, Augusta, GA USA; 4 Oral Biology, Augusta University, Augusta, GA USA; 5 Orthopaedic Surgery, Augusta University, Augusta, GA USA; 6 Pathology and Laboratory Medicine, Medical University of South Carolina, Charleston, SC USA; 7 Institute for Regenerative and Reparative Medicine, Augusta University, Augusta, GA USA; 8 Research Service of the Ralph H Johnson VA Medical Center, Charleston, SC USA; 9 Charlie Norwood VA Medical Center, Augusta, GA USA; 10 Department of Regenerative Medicine and Cellular Biology, Charleston, SC USA; 11 Present Address: Wake Forest Institute for Regenerative Medicine, Wake Forest School of Medicine, Winston-Salem, NC USA; 12 Division of Anatomy, College of Medicine, Ohio State University, Columbus, OH USA

## Abstract

Bone wound healing is a highly dynamic and precisely controlled process through which damaged bone undergoes repair and complete regeneration. External factors can alter this process, leading to delayed or failed bone wound healing. The findings of recent studies suggest that the use of selective serotonin reuptake inhibitors (SSRIs) can reduce bone mass, precipitate osteoporotic fractures and increase the rate of dental implant failure. With 10% of Americans prescribed antidepressants, the potential of SSRIs to impair bone healing may adversely affect millions of patients’ ability to heal after sustaining trauma. Here, we investigate the effect of the SSRI sertraline on bone healing through pre-treatment with (10 mg·kg^-1^ sertraline in drinking water, *n* = 26) or without (control, *n* = 30) SSRI followed by the creation of a 5-mm calvarial defect. Animals were randomized into three surgical groups: (a) empty/sham, (b) implanted with a DermaMatrix scaffold soak-loaded with sterile PBS or (c) DermaMatrix soak-loaded with 542.5 ng BMP2. SSRI exposure continued until sacrifice in the exposed groups at 4 weeks after surgery. Sertraline exposure resulted in decreased bone healing with significant decreases in trabecular thickness, trabecular number and osteoclast dysfunction while significantly increasing mature collagen fiber formation. These findings indicate that sertraline exposure can impair bone wound healing through disruption of bone repair and regeneration while promoting or defaulting to scar formation within the defect site.

## Introduction

Antidepressants, including selective serotonin reuptake inhibitors (SSRIs), are the third most prescribed drug class in the United States. It is estimated that 10% of Americans use antidepressants, representing a greater than 400% increase in use since 1994. Additionally, long-term use is becoming more common; 60% of SSRI users have been on treatment for over 2 years, and 14% of users have been on treatment for more than 10 years.^1^ With this dramatic increase in antidepressant use, comorbidities are now being discovered, including increased risk of fracture and decreased bone mineral densities in adolescents and young adults. Furthermore, in at-risk populations (e.g., post-menopausal women), SSRI use is associated with increased fracture risk, superseeding ^2–5^ other contraindicated drugs, including glucocorticoids^6^ and proton pump inhibitors.^7^

Although there is debate within the field concerning the relative contribution of depression^8,9^ or SSRI use within this paradigm,^10–13^ more recent information suggests that SSRI use by patients without mental health disorders, particularly those with vasomotor disorders, also leads to increased fracture risk, further highlighting a possible link between SSRIs and decreases in bone health.^14^ While clinical correlations between SSRI treatment and negative bone phenotypes have been established, research concerning SSRI use and bone health has only recently been extended to specific effects on bone wound healing, where it was found that fluoxetine negatively affected limb fracture healing.^15^ It follows that if SSRIs can affect bone health and even bone cell activity,^16–19^ use may negatively affect the repair and remodelling process. Therefore, it is important to elucidate the effects of the different SSRI antidepressants on bone healing, as the demographic of SSRI users intersects with patients at increased baseline risk of fracture.

SSRI use presents an additional possible complication to craniofacial bone wound healing. Craniofacial injuries are the leading cause of mortality and morbidity in individuals under 45 years of age,^20,21^ accounting for over 20 million visits to the emergency room each year. The increased long-term use of SSRIs creates a concern for the recovery period after injury or iatrogenic craniofacial surgery,^21,22^ since large defects in the calvarium are known to present with inadequate healing.^23^ Such wounds are already compromised and often require multiple clinical interventions, including grafts and/or growth factor therapies.^24,25^ Further evidence in a recent cohort study found that SSRI use results in higher rates of dental implant failure: 10.6% compared to 4.6% for non-SSRI users.^26^ This is a significant finding, as an estimated 69% of adults between 35 and 44 years of age have lost at least one permanent tooth, and by the age of 74, 26% of adults will have lost all of their permanent dentition.^27,28^ The association between dental implant failure and SSRI use points to impaired osseointegration of the implants, which has a concomitant increase in fracture risk associated with failure.

Due to the current frequent use of SSRIs, these patient populations are at undue risk of potential negative effects on bone healing. In this study, we aim to test the hypothesis that the SSRI drug sertraline impairs acute bone wound healing and/or the quality of the regenerated bone in order to determine if sertraline use has a direct effect on bone healing. We are utilizing an established preclinical calvarial fracture model to test the effects of sertraline use in concurrence with a simulated clinical intervention using the growth factor therapy bone morphogenetic protein 2 (BMP2).^29,30^ These results will provide a first step for clinical translation to vulnerable populations and may inform clinical interventions for fracture healing.

## Results

### Sertraline exposure reduces bone formation

**Fig. 1:**
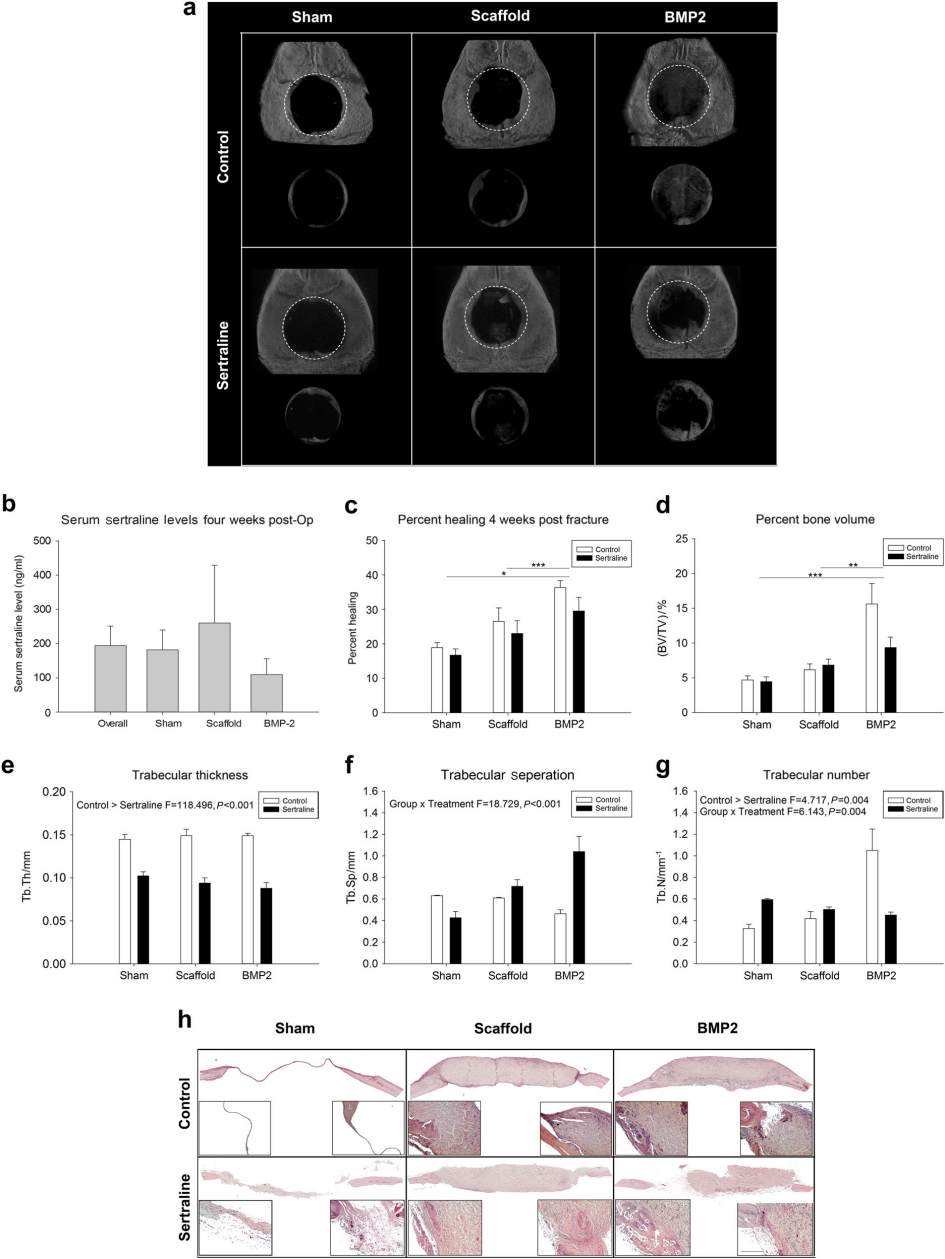
Morphometric parameters of bone within the craniectomy defects 4 weeks post operation. **a** Representative µCT 3D reconstructions of critical-sized mouse calvarial defect. **b** Serum sertraline exposure levels. **c** Radiographic analysis of percent bone healing within the craniectomy defect. BMP2 groups were found to have greater healing, while treatment was not significant (*P* = 0.117). 3D µCT morphometric parameters of bone within the craniectomy defect. **d** BV/TV showed a significant increase in healing in the BMP2 groups. **e** Tb.Th had a significant decrease in the treated groups (*P* < 0.001). **f** Tb.Sp showed a trend for greater trabecular separation but was not statistically significant (*P* = 0.108). **g** Tb.N., the SSRI treatment, was found to significantly decrease trabecular number (*P* = 0.035). **h** H&E histological representatives. Scale bar is 40 µm. *n* = 10 per control sham, PBS, BMP2 and sertraline sham groups; *n* = 7 for sertraline PBS; *n* = 9 for sertraline BMP2. *P < 0.05; **P < 0.01; ***P < 0.001. Data are means ± standard errors

No significant differences in weight were observed due to sertraline exposure (Supplementary Fig. 1). Figure 1a illustrates representative micro-computed tomography (µCT) reconstructions for each group. Analysis of the amount of calvarial defect regeneration and/or mineralization showed that sertraline-exposed animals had reduced bone healing within the defect site at the 4-week post-operative time point. This finding was especially striking in the groups where osteogenesis was driven by administration of the growth factor BMP2. Of the animals exposed to sertraline, the average circulating serum level at sacrifice was (123.42 ± 29.91), mimicking human circulating steady-state levels, and there was no significant difference between surgical groups (*P* = 0.204; Fig. 1b). A natural log transformation was performed to normalize percent healing data for statistical analysis. It was found that BMP2-treated animals had significantly greater healing than the sham and scaffold-only groups (*P* < 0.001 and *P* = 0.05, respectively). Sertraline-exposed animals showed an approximate 10% decrease in percent healing within the defect for the matrix and BMP2-treated groups (Fig. 1c). After a square root transformation, µCT analysis showed that the BV/TV parameter closely aligned with the percent healing, and the BMP2-treated groups had greater bone volume than the sham or scaffold-only groups (*P* < 0.001 and *P* = 0.003, respectively). The increase in BMP2-driven bone volume appeared to be inhibited with exposure to sertraline, but the difference was not statistically significant (*P* = 0.087; Fig. 1d).

Further assessment of the regenerated bone within the defects demonstrated a significant decrease in trabecular thickness (Tb.Th) with sertraline exposure (*P* < 0.001; Fig. 1e). Due to a violation of normality, a rank transformation was performed for the variable trabecular separation (Tb.Sp). A significant interaction term for sertraline exposure by surgical group was observed (*P* < 0.001). This result appears to be due to a large increase in Tb.Sp for the BMP2-treated groups, while no difference was found in the sham or scaffold surgical groups. A trend for greater Tb.Sp for sertraline-exposed animals was demonstrated but was not statistically significant (*P* = 0.108); there was no significant difference by surgical group (*P* = 0.255) (Fig. 1f). However, there was a significant difference in trabecular number (Tb.N) by sertraline exposure, with exposed animals having significantly lower Tb.N (*P* = 0.035; Fig. 1g). To analyse Tb.N, an inverse transformation was performed to allow for homogeneity of variance and normality. There was a significant interaction term for sertraline exposure by surgical group (*P* = 0.004) due to a large increase in Tb.N in the BMP2 group. In comparison, no difference between exposure was found in the sham and scaffold surgical groups. H&E histological representations of the reduced healing in sertraline-exposed animals can be found in Fig. 1h (and Supplementary Fig. 2).

### Sertraline promotes scar formation

**Fig. 2:**
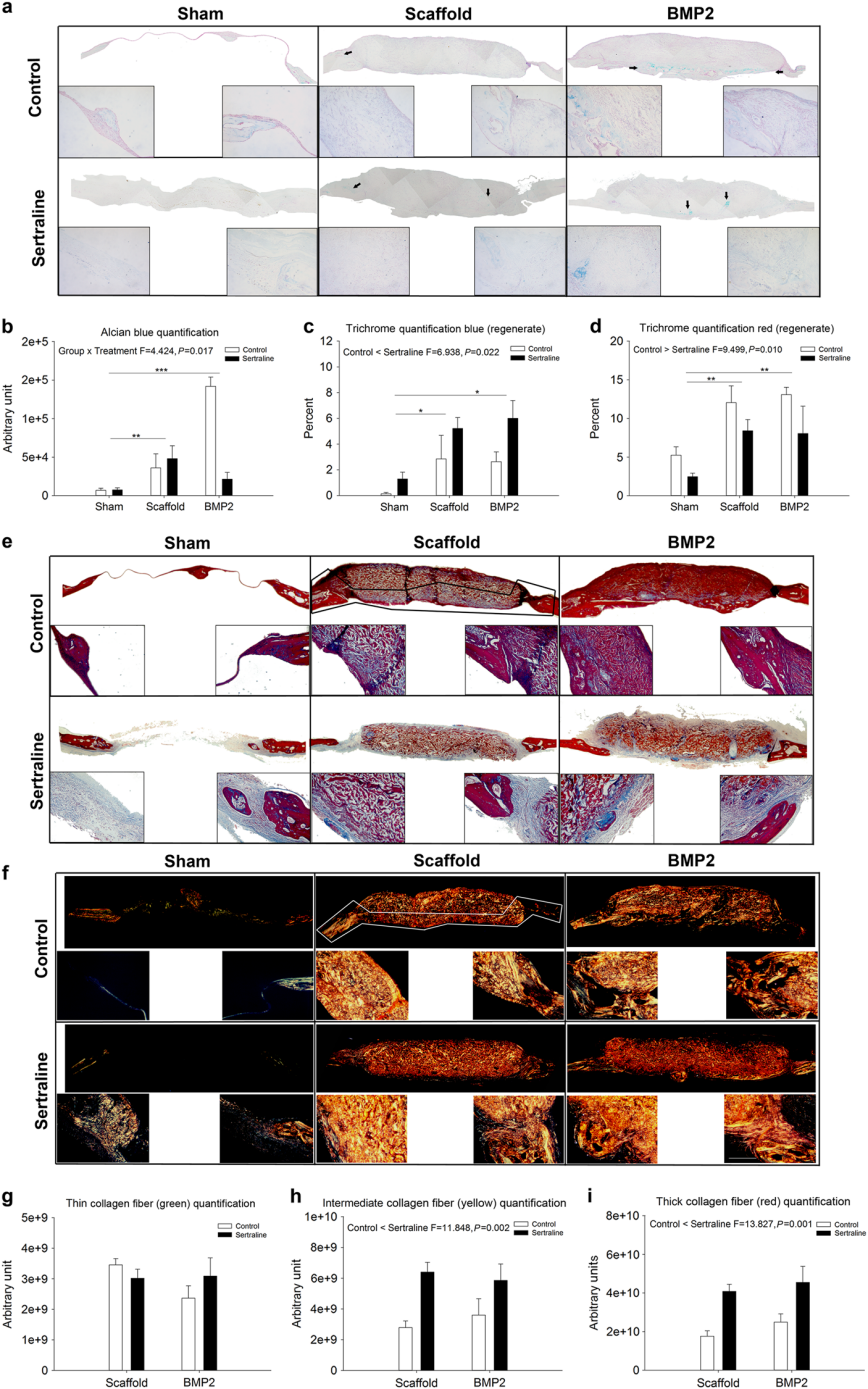
Histological analysis of the critical-sized mouse calvarial defects at 4 weeks post surgery. **a** Representative Alcian Blue images of the entire defect site for control and SSRI-exposed sham, scaffold, and BMP2 surgical groups. **b** Quantification of cartilage within the defect site showing a significant interaction effect between the treatment and surgical groups (*P* = 0.017). The sertraline-exposed scaffold and BMP2 animals were found to have increased cartilage formation compared to sham (*P* < 0.01 and *P* < 0.001, respectively). **c** Quantification of the collagen fibers within the regenerate contained in the defect site. Sertraline-exposed animals contained significantly more collagen fibers (*P* = 0.022), with both the scaffold (*P* = 0.028) and BMP2 surgical groups (*P* = 0.017) having increased collagen within the regenerate compared to sham for both groups. **d** Quantification of bone within the regenerate contained in the defect site. Sertraline-exposed animals contained significantly decreased bone (*P* = 0.010), while the scaffold and BMP2 surgical groups had increased bone within the regenerate compared to sham (*P* = 0.003). **e** Representative Masson’s Trichrome images of the entire defect site for control and sertraline-exposed sham, scaffold, and BMP2 surgical groups. **f** Representative-polarized Picrosirius Red images of the entire defect site for control and sertraline-exposed sham, scaffold, and BMP2 surgical groups. **g** Quantification of the immature (green) collagen fibers within the site. **h** Quantification of medium thickness (yellow) collagen fibres within the regenerate, indicating that sertraline-exposed animals had increased numbers of maturing collagen fibres (*P* = 0.002). **i** Quantification of mature, thick (red) collagen fibers within the regenerate, showing that sertraline-exposed animals had increased mature, thick collagen fibers (*P* = 0.001). Black arrows indicate cartilage formation, and black/white outlines indicate the regenerate analysed. Higher magnification images were taken of the endocranial (left) and surgical margin (right). Scale bar is 40 µm; *n* = 3 per group; *P < 0.05; **P < 0.01; ***P < 0.001. Data are means ± standard errors

Histological analysis of the defect site 4 weeks post-operatively showed more cartilage in the scaffold and BMP2 surgical groups for both the control and sertraline-exposed animals (Fig. 2a, b and Supplementary Fig. 3). However, sertraline appeared to attenuate the ability of BMP2-treated animals to form cartilage. Masson’s Trichrome analysis of the regenerates showed that sertraline-exposed animals contained significantly more collagen fibers (*P* = 0.022), with both the scaffold (*P* = 0.028) and BMP2 surgical groups (*P* = 0.017) having more collagen within the regenerate than in the sham group for both groups. Consequently, the sertraline-exposed animals contained a significantly decreased amount of osteoid (*P* = 0.010), while the scaffold and BMP2 surgical groups had increased osteoid formation within the regenerate compared with the sham group (*P* = 0.003; Fig. 2c–e and Supplementary Figure 4). To further characterize the collagen fibers within the defect, polarized Picrosirius red images were analysed (Fig. 2f and Supplementary Fig. 5). Quantification of the immature, thin (green; Fig. 2g), medium thickness (yellow; Fig. 2h) and mature, thick (red; Fig. 2i) collagen fibers within the regenerate indicated that sertraline-exposed animals had increased yellow and red collagen fibers (*P* = 0.002 and *P* = 0.001, respectively) compared to the unexposed animals. Sham groups were excluded from analysis due to a lack of detectable polarized fibers for either the control or sertraline-exposed groups.

### Sertraline exposure alters cell viability and function

**Fig. 3:**
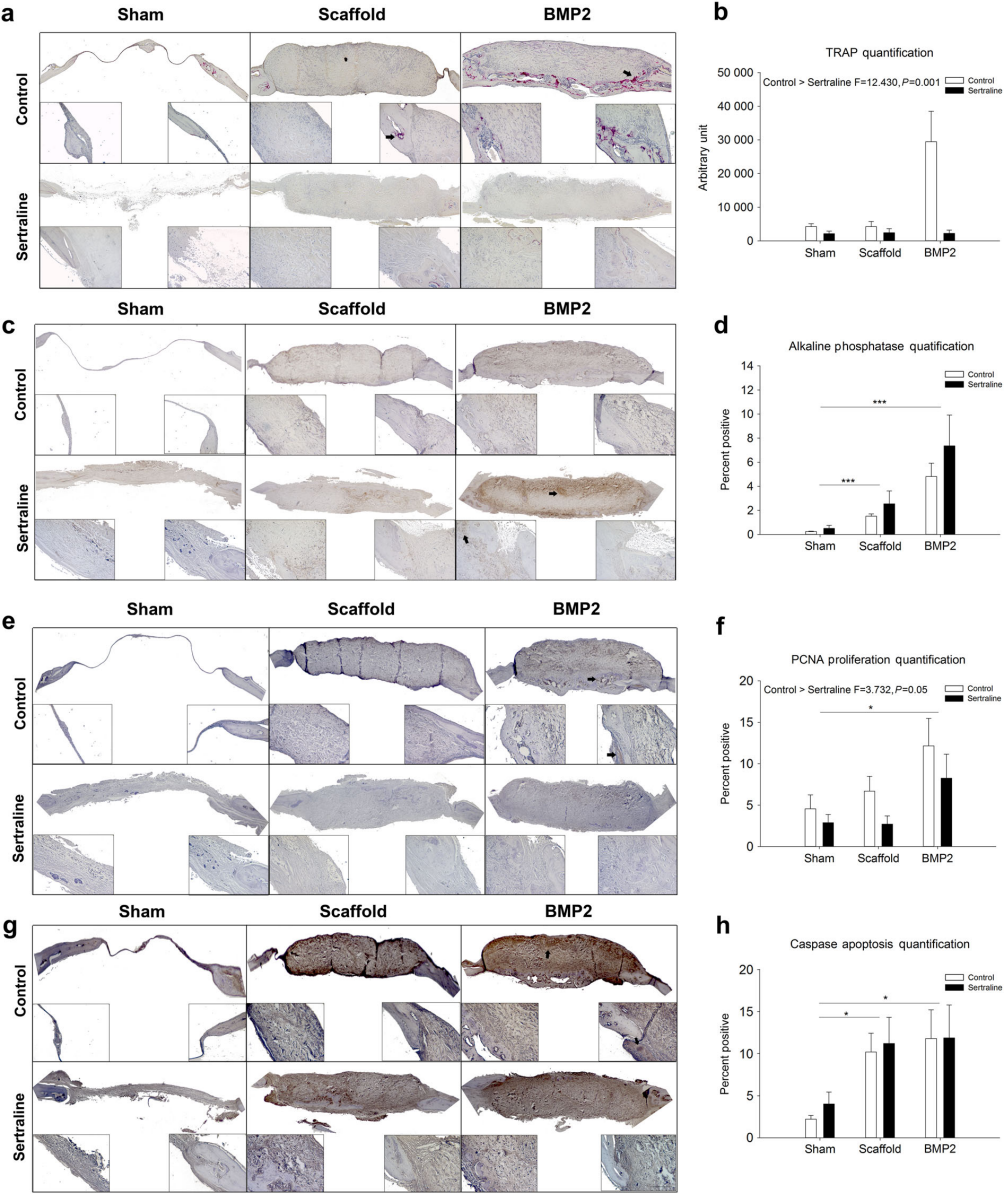
Immunohistochemical analysis of the critical-sized mouse calvarial defects 4 weeks post surgery. **a** TRAP images of the entire defect site for control and sertraline-exposed sham, scaffold, and BMP2 surgical groups. **b** Quantification of the number of TRAP-positive osteoclasts within the defect, which indicates that sertraline exposure decreases osteoclast activity (*P* = 0.001). **c** Alkaline phosphatase immunohistochemistry photomicrographs of control and sertraline-exposed sham, scaffold, and BMP2 surgical groups. **d** Quantification of the alkaline phosphatase-positive cells within the defect, demonstrating that the exposed scaffold and BMP2 groups had increased levels of alkaline phosphatase activity compared to the sham group (*P* < 0.001). **e** Proliferating cell nuclear antigen photomicrographs of control and sertraline-exposed sham, scaffold, and BMP2 surgical groups. **f** Quantification of the PCNA-positive cells within the defect, showing that the exposed animals had decreased proliferation (*P* = 0.05) compared to the control animals, while the exposed BMP2 group had increased levels of PCNA activity compared to the sham group (*P* < 0.05). **g** Caspase immunohistochemistry photomicrographs of control and sertraline-exposed sham, scaffold, and BMP2 surgical groups. **h** Quantification of the caspase-positive cells within the defect, indicating that the exposed scaffold and BMP2 groups had increased levels of apoptosis compared to the sham group (*P* < 0.05). Black arrows indicate examples of positive staining. Higher magnification images were taken of the endocranial (left) and surgical margin (right). Scale bar is 40 µm; *n* = 3 per group; *P < 0.05; ***P < 0.001. Data are means ± standard errors

In order to determine if sertraline exposure promoted scar formation instead of bone remodelling due to negative effects on osteoclast (TRAP) or osteoblast (ALP) activity, immunohistochemical analyses were performed. Sertraline exposure was found to significantly diminish TRAP activity (*P* = 0.001; Fig. 3a, b and Supplementary Fig. 6). While ALP levels showed no significant difference between the control and sertraline-exposed groups, a significant increase was observed in the sertraline-exposed scaffold and BMP2 groups compared to the sham group (*P* = 0.001; Fig. 3c, d and Supplementary Fig. 7). To elucidate if differences in the ability of the sertraline-exposed animals to heal were due to changes in cellular proliferation (PCNA; Fig. 3e and Supplementary Fig. 8) or apoptosis (Caspase; Fig. 3g and Supplementary Fig. 9), IHC was performed. Cellular proliferation was shown to be significantly increased (*P* = 0.05; Fig. 3f) in the control animals, while no difference in apoptosis was observed (Fig. 3h).

### Sertraline treatment elicits a cellular response in MC3T3-E1s pre-osteoblast isotype cells

**Fig. 4:**
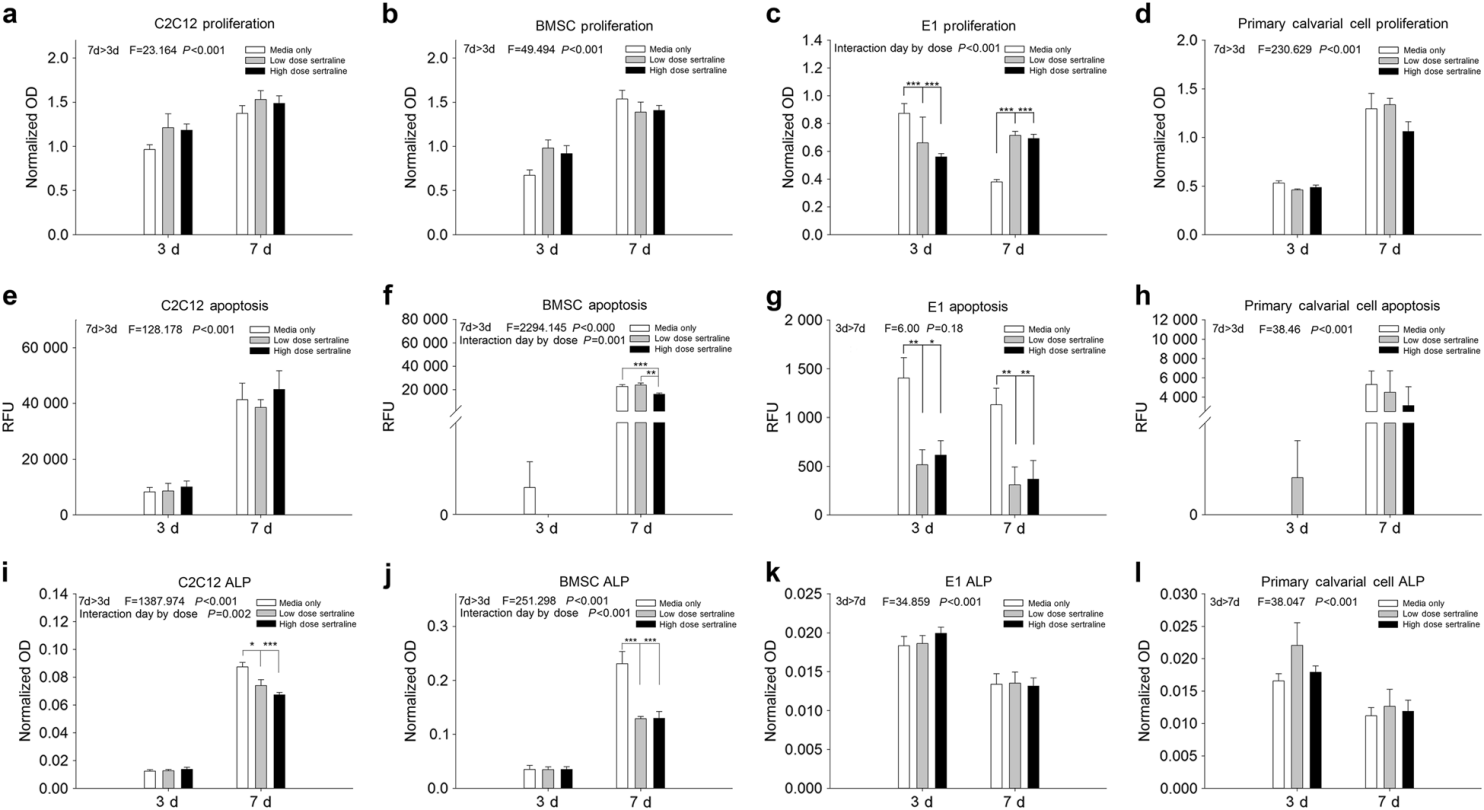
Effect of sertraline treatment on the function of calvarial cell populations. **a**–**d** Effect of sertraline on the proliferative potential of cell types within the defect site. **e**–**h** Apoptotic effect of sertraline. **i**–**l** ALP function of cell types within the defect site after sertraline treatment. **b** Pre-osteoblast E1s were shown to have significantly decreased proliferation after sertraline treatment for 3 days, while by 7 days, a significant increase was observed (*P* < 0.001). In comparison, **e**–**h** the apoptotic effect of sertraline was shown to significantly decrease for **f** BMSCs (*P* < 0.01) and **g** E1s (*P* < 0.01), with **e** C2C12s and **h** primary calvarial cells also showing trends toward decreased apoptosis with sertraline treatment compared to control. Sertraline was found to significantly decrease **i** C2C12 (*P* < 0.001) and **j** BMSC (*P* < 0.001) ALP production at 7 days. *n* = 2–6 per cell type. ^#^ different from control; * different from low-dose sertraline; *P < 0.05; **P < 0.01. Data are means ± standard errors

To determine the direct effect of sertraline treatment on the function of specific cell types contained within the calvaria, C2C12 (fibroblast phenotype), BMSCs (bone marrow-derived stem cells), E1s (mouse calvarial pre-osteoblasts), and primary calvarial cells were treated with control (no dose), low, and high doses of sertraline. E1 cells were shown to have a varied response to sertraline treatment between 3 and 7 days, with decreased proliferation at 3 days and a significant increase in proliferation by 7 days compared to controls (*P* < 0.001 for both; Fig. 4c). Additionally, sertraline treatment was shown to protect against apoptosis for BMSCs (*P* < 0.01; Fig. 4f) and E1s (*P* < 0.05; Fig. 4g) by 7 days. Primary calvarial cells also showed decreased apoptosis compared to controls; however, no significant differences were observed (Fig. 4h). Finally, the effect of sertraline on ALP production for each of the cell types within the calvaria was found to be significantly decreased for both C2C12 and BMSCs at 7 days (*P* < 0.001; Fig. 4j, k), while no effect was observed for E1s or primary cells.

### Sertraline treatment modulates collagen and serotonin-related factors

**Fig. 5:**
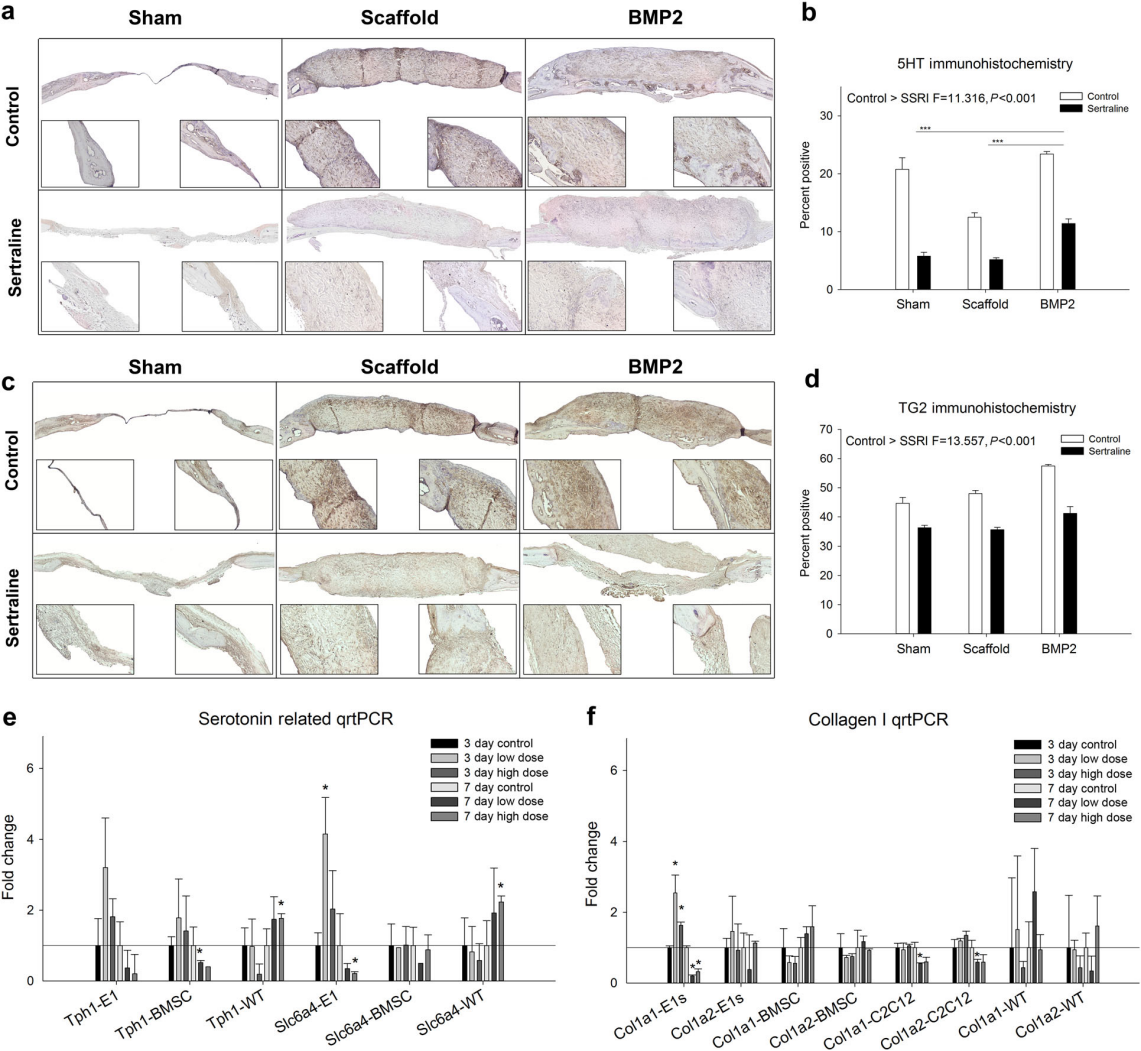
Immunohistochemical analysis of serotonin (5-hydroxytryptamine, 5HT) and tissue transglutaminase (TG2) levels in mouse critical-sized calvarial defects 4 weeks post surgery. **a** 5-Hydroxytrptamine (5HT) immunohistochemistry photomicrographs of control and sertraline-exposed sham, scaffold, and BMP-2 surgical groups. **b** Quantification of the 5HT-positive cells within the defect. Sertraline exposure results in a significant decrease in serotonin within the defect when compared with untreated control samples (*P* < 0.001), and the BMP-2-treated groups showed significantly more 5HT positivity compared to both the sham and scaffold groups (*P* < 0.001). **c** Tissue transglutaminase (TG2) immunohistochemistry photomicrographs of control and sertraline-exposed sham, scaffold, and BMP-2 surgical groups. **d** Quantification of the TG2-positive cells within the defect. Sertraline exposure resulted in a significant decrease in TG2 levels within the defect when compared to untreated control samples (*P* < 0.001). Higher magnification images were taken of the endocranial (left) and surgical margin (right). **e** Gene expression of *Tph1* and *Slc6a4* showed downregulation at 7 days with sertraline treatment in E1 and BMSC cells, while WT cells showed a significant upregulation in these genes at 7 days with high-dose sertraline treatment (*P* < 0.05). C2C12 cells did not amplify either gene. **f** E1 and C2C12 cells showed a significant downregulation of *Col1a1* and *Col1a2* at 7 days with sertraline treatment (*P* < 0.05). *n* = 3 per group; *P < 0.05; ***P < 0.001. Data are means ± standard errors

To investigate the potential mechanism by which sertraline treatment impairs healing, histological analysis on the bioavailability of serotonin (5HT) and its transporter, TG2 (tissue transglutaminase), within the defect site was performed (Fig. 5a, c and Supplementary Figs. 10–11). Control animals had significantly higher levels of 5HT within the defect site compared to exposed animals (*P* < 0.001; Fig. 5b). The levels of TG2 did not differ significantly between the exposed and non-exposed animals; however, there was an observed decrease in TG2 with sertraline exposure (Fig. 5d). Analysis of the gene expression of the enzymes involved in serotonin metabolism (*Tph1* and *Tph2* or tryptophan hydroxylase) and the cell surface receptor of serotonin (*Slc6a4* or solute carrier family 6 member 4) in both isotype cells and isolated calvarial WT cells showed that *Tph2* was not present in any of the cell types, and C2C12 cells did not amplify *Tph1, Tph2* or *Slc6a4*. However, at 7 days, E1s and BMSCs showed downregulation of *Tph1*, while WT cells showed significant upregulation with high-dose sertraline. Following the same pattern, E1 and BMSC cells had downregulated *Slc6a4* with sertraline treatment, while there was an observed upregulation in WT cells at 7 days. To determine if sertraline treatment affected collagen gene expression, *Col1a1* and *Col1a2* (genes encoding the pro-alpha chains) were investigated in our cell types. We found that E1s significantly downregulated *Co1a1* and that C2C12 cells significantly downregulated both *Col1a1* and *Col1a2* at 7 days with low- and high-dose sertraline treatment (*P* < 0.05). BMSC and WT cells showed no significant difference in the gene expression of *Col1a1* or *Col1a2* with sertraline treatment.

## Discussion

While evidence suggests that SSRIs have the ability to increase rates of osteoporotic fracture and dental implant failure rates, the direct effect of sertraline treatment on bone healing has yet to be elucidated. This study showed that sertraline exposure decreased bone healing with significant declines in Tb.Th and Tb.N, independent of specific circulating serum sertraline levels, suggesting a possible on/off mechanism of action for sertraline’s effect on bone healing instead of a dose-dependent effect. While increased Tb.Sp coupled with significantly decreased Tb.N and Tb.Th after sertraline exposure support the decreased percent healing and bone volume observed in the sertraline-exposed groups, these significant differences did not lead to a corresponding significant decline in either factor. This disparity seems to indicate that, while the overall bone volume was not significantly affected by sertraline, the organization of the regenerated bone was severely hampered, with sertraline exposure causing the regenerating bone to be organized in thin, widely spaced trabeculae. Subsequently, histological analysis of the regenerate within the defect site showed increased amounts of disjoined woven bone with a lack of continuous bone regrowth after sertraline exposure. Bone organized in such a fashion would be unlikely to support a load, increasing the mobility within the defect site and therefore requiring an alternative healing pathway to accommodate such displacement.

Due to this disruption of normal bone healing, we hypothesized that sertraline drives scar formation (repair rather than regeneration) within the defect site. Decreased cartilage formation coupled with increased mature collagen fibre formation in the sertraline-exposed animals would alter the secondary fracture healing process, resulting in decreased bone formation. The significant presence of medium and thick collagen fibres within the defect show a mature collagen structure in the exposed animals, indicating that the innate healing response was sufficient to form repair tissue (scar) but was not sufficient to produce mature bone. This result is not indicative of delayed healing due to a lack of difference in the amount of thin, immature collagen fibre formation between the control and sertraline-exposed animals. Interestingly, the delivery of the osteoinductive morphogen, BMP2, previously shown to accelerate healing through endochondral ossification (cartilage formation) in this model,^29–31^ was insufficient to overcome the effects of sertraline for either cartilage or subsequent bone formation. The decreases in bone formation in addition to the significant increase in mature collagen fibres found within the defect site indicate disruption of bone regeneration leading to scar formation as a repair mechanism.

We found that the observed decrease in bone healing during sertraline exposure was partially due to the negative effects of the sertraline on osteoclast function. Significant declines in TRAP-positive cells within the defect site indicate that the sertraline decreased osteoclast function, thereby reducing the ability of bone to regenerate due to reduced removal of fractured and dead bone within the defect site. As there was no difference in the rate of apoptosis in treated animals, the lack of TRAP-positive cells is not likely due to sertraline-driven apoptosis of osteoclasts at this time point, but perhaps is a direct effect on osteoclast function. Ortuno et al. demonstrated that fluoxetine treatment inhibits osteoclast differentiation and function through a Ca^2+^-mediated pathway.^32^

Our cell cultures also corroborate this paradigm, showing decreased apoptosis and increased proliferation for the pre-osteoblast population treated with sertraline. These findings differ from the results of a recent study by Bradaschia-Correa et al., who found that fluoxetine treatment inhibited proliferation, osteoblast differentiation and mineralization within an appendicular skeletal defect model.^15^ These contrasting findings could be attributed to varying mechanisms of action between the SSRI drugs or to the fact that cells from the cranium and the appendicular skeleton differ in origin, thereby affecting their response to extrinsic factors. While sertraline was not shown to have a direct negative effect on the function or viability of osteoblasts, the ability of the cells to create mineralized bone is dependent on an organized matrix, which is lacking in the sertraline-exposed animals. This uncoupling of osteoclast/osteoblast function could explain the lack of organization of the regenerate within the defect site of treated animals.

The concept that serotonin may play a direct role in bone health is not a new one,^33–37^ as research has examined the neural, gut, and skeletal health-related effects of serotonin. The focus has been placed on the presence and modulation of serotonin receptors, the serotonin transporter and direct and indirect effects of serotonin-modulating drugs, SSRIs, on bone and bone cells.^38,39^ Little resolution for these effects has been reached. Not surprisingly, the presence of serotonin can certainly affect the skeleton through proposed bone-related molecular pathways, including WNT^40–43^ and Notch.^44,45^ Furthermore, both positive and negative effects on bone cells have been observed, suggesting that homoeostasis of this amino acid derivative is key. Alterations, particularly through pharmacological targeting of serotonin synthesis, may result in changes to the hard tissues in both normal remodelling processes as well as in healing.

Overall, the mechanism by which sertraline disrupts bone health and regeneration remains largely unknown. To try and elucidate if sertraline affects healing through direct modulation of serotonin within the fracture site, we investigated if serotonin levels were altered with sertraline treatment. We found that 5HT levels were significantly depleted with treatment, but TG2 serotonin transporter levels, while modulated, were not significantly downregulated. The maintenance of TG2 levels could be due to TG2 being involved in the transportation of other molecules;^46^ therefore, the reduction of one molecule, serotonin, would not substantially affect TG2 levels. Further investigation into the expression levels of serotonin-related genes showed that none of the cell types utilized in this study amplified *Thp2*, the neuronal tryptophan hydroxylase.^47^ However, it was found that E1 cells showed a downregulation of both *Tph1* and *Slc6a4* at 7 days with sertraline treatment, indicating that sertraline may modulate the ability of pre-osteoblasts to synthesize serotonin or respond to extracellular serotonin through its cell surface receptor. WT cells showed a significant increase in both *Tph1* and *Slc6a4* at 7 days with sertraline treatment. The difference between the WT and E1 cellular response to sertraline treatment could be due to the WT population having a variety of cell types within it that respond to sertraline treatment by upregulating these genes. Interestingly, C2C12 cells did not amplify any of the serotonin-related genes, indicating that C2C12 cells might be involved in serotonin signalling pathways. Due to the observed disorganization of the collagen matrix within the sertraline-exposed animals, we investigated the *Col1a1* and *Col1a2* genes to determine if sertraline treatment directly affects the ability of E1, C2C12, BMSC or WT cells to produce collagen. We found that treatment reduced the expression levels of both of these genes at 7 days with low- and high-dose sertraline treatment for E1 and C2C12 cell types. The downregulation of these genes could explain the inhibited bone remodelling that occurred in our fracture model despite evidence that osteoblasts had enhanced ALP activity with sertraline exposure. The inhibition of these cells' capacity to produce collagen and therefore an organized matrix would hamper the ability of osteoblasts to lay down mineralized bone.

In conclusion, our study suggests that sertraline exposure alters bone healing largely by enhancing collagen formation while subsequently hampering cartilage formation and osteoclast function that would facilitate normal bone formation. Of the resulting bone contained within the defect, the organization and structure are negatively impacted, indicating the compromised quality of the remodelled bone with sertraline treatment. As use of SSRIs continues to increase globally, this study provides important preclinical information concerning the disruptive effects of sertraline treatment on bone wound healing. These data suggest that there is a susceptible population of patients with impaired bone health due to pharmacological intervention for depression once thought to only include patients with pre-existing compromised bone (i.e., osteoporosis).^2–5^ Although this topic will remain controversial, the increase in reports of interaction effects between bone health and SSRI use^14,26^ is revealing a potentially larger problem not limited to osteoporosis. Further study is needed to determine if long-term SSRI treatment alters bone remodelling through other pathways such as TNF-α signalling linked to inflammation and early healing. Such alteration may require modification of treatment following bone injury or in anticipation of surgical bone defect repair.

## Materials and methods

### Animals

Eight-week-old C57BL6 male mice (Jackson Laboratory, Bar Harbor, ME, USA) were treated with 10 mg·kg^-1^ sertraline (InvaGen, Hauppauge, NY) in drinking water (*n* = 26) or normal drinking water (*n* = 30) for 2 weeks prior to the surgeries, and treatment was continued until killing. The animals were randomized into three surgical groups: (a) empty defect/sham to show normal bone healing with no surgical intervention; (b) implanted with 4-mm DermaMatrix scaffold (Synthes, West Chester, PA, USA; *n* = 20) soak-loaded with sterile PBS to elucidate if the matrix itself has osteoconductive properties; or (c) 4-mm DermaMatrix scaffold soak-loaded with 542.5 ng BMP2 (PeproTech, Rocky Hill, NJ, USA; *n* = 18) to represent the clinical intervention. A critical-sized calvarial defect was then performed as previously described.^29–31^ Briefly, the mice were anesthetized with isoflurane (Bethlehem, PA, USA), and a midline scalp incision was used to expose and remove the periosteum. A 5-mm craniectomy defect was trephinated using a slow-speed hand drill. Each craniectomy defect was filled with the assigned treatment above. The incision was then sutured closed with 6 × 0 polypropylene suture.

The animals were weighed and monitored 2 days post surgery for any signs of pain or distress, with daily monitoring continuing until the time of sacrifice. The mice were sacrificed at 4 weeks post surgery, at which point the skulls were collected and subjected to radiography, micro-computed tomography (µCT) and histological analysis. Serum was also collected at the time of  sacrifice for systemic sertraline detection. Sacrifice was performed by asphyxiation using CO_2_ gas followed by exsanguination. All procedures were carried out with the approval of the Augusta University and Medical University of South Carolina IACUC, in an Association for Assessment and Accreditation of Laboratory Animal Care International accredited facility, where all husbandry and related services are provided by the Division of Laboratory Animal Resources. All procedures and the reporting thereof are in compliance with the Animal Research: Reporting in Vivo Experiments (ARRIVE) guidelines.^48^

### Sertraline ELISA

Sertraline levels were determined from serum collected from all specimens in duplicate following the manufacturer’s instructions (Sertraline forensic (RTU) kit, Neogen, KY, USA).

### Radiography

Calvariae were isolated and bisected from the occipital protuberance to the nasal cavity, followed by radiography to assess percent bone healing, as previously described.^29^ Briefly, calvarial specimens were radiographed using a Faxitron X-Ray imaging instrument (Faxitron X-Ray, Wheeling, IL, USA) and PPL film (Carestream, NY, USA) following initial calibration. Percent bone healing was estimated utilizing a 5.0-mm region of interest (ROI). Using ImageJ software (NIH, Washington, DC, USA), each 5.0-mm ROI was isolated and subjected to system default binary thresholding. After thresholding, the ROI was analysed for the amount of new bone and percent bone healing was calculated relative to the area of a 5.0-mm circle.

### Micro-computed tomography

Micro-computed tomography (µCT) analysis was performed as described previously.^29,30^ Briefly, µCT images of the calvarial specimens were obtained via ex vivo µCT systems (µCT40; Scanco Medical, Bruttisellen, Switzerland at 70kVp, 0.1 mA, and Skyscan 1174; Skyscan, Aartlesaar, Belgium at 50 kVp and 0.8 mA), each with a 0.25-mm aluminium filter. Of note, the machines were cross-calibrated with manufacturer-provided hydroxyapatite phantoms and sample bones for consistency, but no density measures were abstracted. The calvariae were placed in a plastic sample holder with the sagittal suture oriented parallel to the image plane and scanned in air or PBS at 13-µm isotropic voxels, 200–1 300-ms integration time, 0.5° rotation step and frame averaging of 4. All scans were then reconstructed using NRecon software (Skyscan) with the same reconstruction parameters (ring artefact reduction of 5, beam hardening correction of 20%). For 3-D analysis (CTAn software, Skyscan), a gauss filter at 1.0-pixel radius and a global threshold range of 50–140 was used. This segmentation approach allowed viewing of the normal bone architecture in the binary images as seen in the original reconstructed images.^49^) All reconstructed images were adjusted to this grey scale before running the 3D analysis. Standard 3D morphometric parameters,^49^ including percent bone volume, trabecular number, trabecular thickness and trabecular separation, were determined in an ROI (5.0-mm circle, 100 cuts = 1.3 mm, total volume = 25.5 mm^3^). Representative 3D images were created using CTvox software (Skyscan).

### Histological analysis

After µCT scanning, representative samples (*n* = 3) from each group (sham, scaffold and BMP2 for control and sertraline-treated animals) were placed in 3.7% formaldehyde for 2 days, stored in 70% ethanol and then decalcified in 0.25 mol·L^−1^ EDTA at pH 7.4 for 10 days. Samples were then washed, dehydrated in graded ethanol (70%–100%), cleared in xylene and embedded in paraffin. Histology and immunohistochemistry were performed on three 7-µm sections at least 30 μm apart per sample for analysis of the defect area. Sections were stained with Alcian Blue, Masson’s Trichrome (MTC; Thermo Scientific, Waltham, MA USA), Picrosirius Red (PSR), and tartrate-resistant acid phosphatase (TRAP; Sigma) by standard methods. Stained sections were photographed using a Motic Inverted Microscope with attached camera (Motic, BC, Canada), and the total area of each stain within the defect site was quantified using Visiopharm software (Visiopharm, Broomfield, CO). Alcian Blue sections were analysed for total area of cartilage (blue); MTC osteoid (red) and collagen tissue (blue); PSR for thin, immature (green), mid-range (yellow), and thick, mature (red) collagen fibres; and TRAP for TRAP-positive osteoclasts (red) within the defect site. MTC and PSR sections were analysed using a region of interest (ROI) isolating the endocranial side of the defect continuous with the surgical margin to better capture the regenerate while excluding the DermaMatrix scaffold.

Immunohistochemistry representative samples (*n* = 3) were incubated with the following primary antibodies: PCNA, a proliferation marker (AbCam, Cambridge, MA, ab18197, 1:3 000); ALP, an osteoblast marker (AbCam ab108337, 1:250); Active Caspase 3, an apoptosis marker (Ab2302, 1:75); tissue transglutaminase, a serotonin transporter (TG2; Covalab, Villeurbanne, France, PAB0024, 1:100); and 5-hydroxytryptamine, serotonin (5HT; LSBio, Seattle, WA, USA LS-B7118, 1:100). Samples were compared at the defect site for percent positivity of the targets of interest using ImageJ Software and the IHC Profiler Open Source Plugin for automated scoring of immunohistochemical staining.^50^

### Cell culture and cell proliferation, apoptosis, and ALP assays

Primary, wild-type, calvarial cells^51^, murine bone marrow stem cells (BMSCs)^52^, as well as isotype cell line myofibroblast C2C12 (ATCC, USA) and pre-osteoblasts MC3T3-E1 (E1; ATCC, USA) were cultured at 37 °C in a humidified 5% CO_2_ incubator. E1 cells were cultured in Alpha Modified Eagle’s Medium (αMEM; Lonza, USA), while the remaining cells were cultured in DMEM (Lonza, USA) containing 10% foetal bovine serum (FBS; Atlanta Biologics, USA), 1% penicillin/streptomycin (penstrep; Lonza, USA), and 0.2% amphotericin B (Lonza, USA) with media changes twice weekly until 95% confluence was reached. At confluence, cells were seeded at a density of 4 000 cells per well for cell function assays. Cells were treated for 3 or 7 days with control (culture) media or with media containing low-level sertraline (34.2 ng·mL^-1^) or high-level sertraline (342 ng·mL^-1^), correlating to human steady-state levels^53^. Cell viability (proliferation) was assessed with the MTS assay (Promega, USA), and cell apoptosis was assessed with the Caspase 3/7 assay (Promega, USA). Quantitative ALP activity was assessed by lysing cells in a 0.1% Triton X lysis buffer, and the whole cell lysis was measured using a SigmaFast p-Nitrophenyl phosphate kit (Sigma-Aldrich, USA) by adding para-nitrophenylphosphate (pNPP) as a substrate assay buffer containing MgCl_2_ for 30 min and reading the kinetics of absorbance at 405 nm on a Gen5 plate reader (BioTek, Winooski, VT, USA).

### Quantitative real-time polymerase chain reaction (qRT-PCR)

Total cellular RNA was extracted using a Qiagen RNeasy mini kit (Qiagen, Valencia, CA, USA), and reverse transcription of RNA was performed using a cDNA Synthesis Kit (Thermo Fisher Scientific, Pittsburgh, PA, USA) according to the manufacturer’s instructions. cDNA was subjected to quantitative PCR using Applied Biosystems TaqMan Gene Expression Master Mix and the TaqMan primers for *Col1a1*(Mm00801666_g1), *Col1a2* (Mm00483888_m1), *Tph1* (Mm01202614_m1), *Tph2* (Mm00557715_m1) and *Slc6a4* (Mm00439391_m1). Data were normalized to *18S* (Mm03928990_g1) ribosomal RNA expression by ΔCT. Quantitative data were compared for gene expression change due to treatment by the ΔΔCT methodology. Statistical differences in gene expression after sertraline treatment were determined as previously published^54^).

### Statistics

Standard *t*-test and two-way ANOVA to investigate the interaction and main effects with post hoc Bonferroni analyses were conducted where appropriate. Violations of homogeneity of variance resulted in the use of Welch’s correction. Violations of normality resulted in the indicated transformation or use of non-parametric alternative tests. Differences were considered significant if *P* ≤ 0.05. Data are means ± standard errors.

## Electronic supplementary material

Supporting Figure Legend

Supplemental Figure 1

Supplementary Figure 2

Supplementary Figure 3

Supplementary Figure 4

Supplementary Figure 5

Supplementary Figure 6

Supplementary Figure 7

Supplementary Figure 8

Supplementary Figure 9

Supplementary Figure 10

Supplementary Figure 11
