# Supplementary material for: Selective serotonin re-uptake inhibitor sertraline inhibits bone healing in a calvarial defect model
Source: Int J Oral Sci. 2018 Sep 3;10(3):25. doi: 10.1038/s41368-018-0026-x (PMC6119683; doi:10.1038/s41368-018-0026-x)
Supplement: Supplementary file 1 — Supporting Figure Legend [file 41368_2018_26_MOESM1_ESM.docx]

**Supporting Figure Legend**

Supporting Information Figure 1: Animal weights. A) Weights of treated versus untreated animals by surgical group. A two-way ANOVA showed a significant increase in weight of the SSRI treated groups, p<0.001, but no difference by surgical group, p=0.537. B) Weight of SSRI treated animals at time of surgery and sacrifice. Paired subjects t-test indicated a significant increase in weight at sacrifice, p<0.001. Data are means ± SE. These results indicate increased weight due to sertraline exposure, consistent with clinical observations of weight gain in patients due to SSRI treatment.

Supporting Information Figure 2: Higher Resolution H&E Stain Corresponding to Figure 1h.

Supporting Information Figure 3: Higher Resolution Alcian Blue Corresponding to Figure 2a,b.

Supporting Information Figure 4: Higher Resolution Masson’s Trichrome Corresponding to Figure 2c.

Supporting Information Figure 5: Higher Resolution PicroSirius Red Corresponding to Figure 2f.

Supporting Information Figure 6: Higher Resolution TRAP Corresponding to Figure 3a,b.

Supporting Information Figure 7: Higher Resolution ALP Immuno Corresponding to Figure 3c,d.

Supporting Information Figure 8: Higher Resolution PCNA Immuno Corresponding to Figure 3e.

Supporting Information Figure 9: Higher Resolution Caspase Immuno Corresponding to Figure 3g.

Supporting Information Figure 10: Higher Resolution 5HT Immuno Corresponding to Figure5 a.

Supporting Information Figure 11: Higher Resolution TG2 Immuno Corresponding to Figure 5c.
