# Supplementary figures and images for: Selective serotonin re-uptake inhibitor sertraline inhibits bone healing in a calvarial defect model
Source: Int J Oral Sci. 2018 Sep 3;10(3):25. doi: 10.1038/s41368-018-0026-x (PMC6119683; doi:10.1038/s41368-018-0026-x)

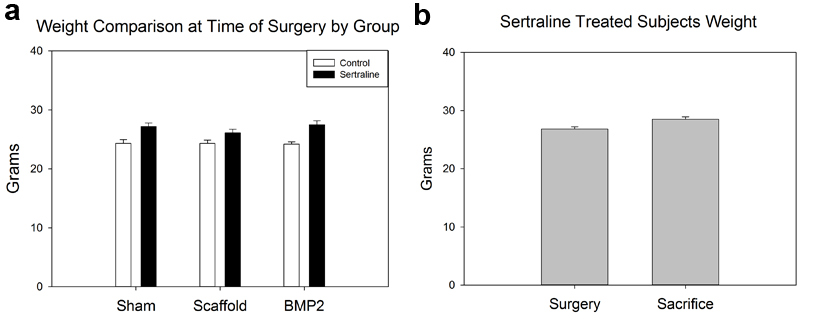

Supplement: Supplementary file 2 — Supplemental Figure 1 [file 41368_2018_26_MOESM2_ESM.tif]

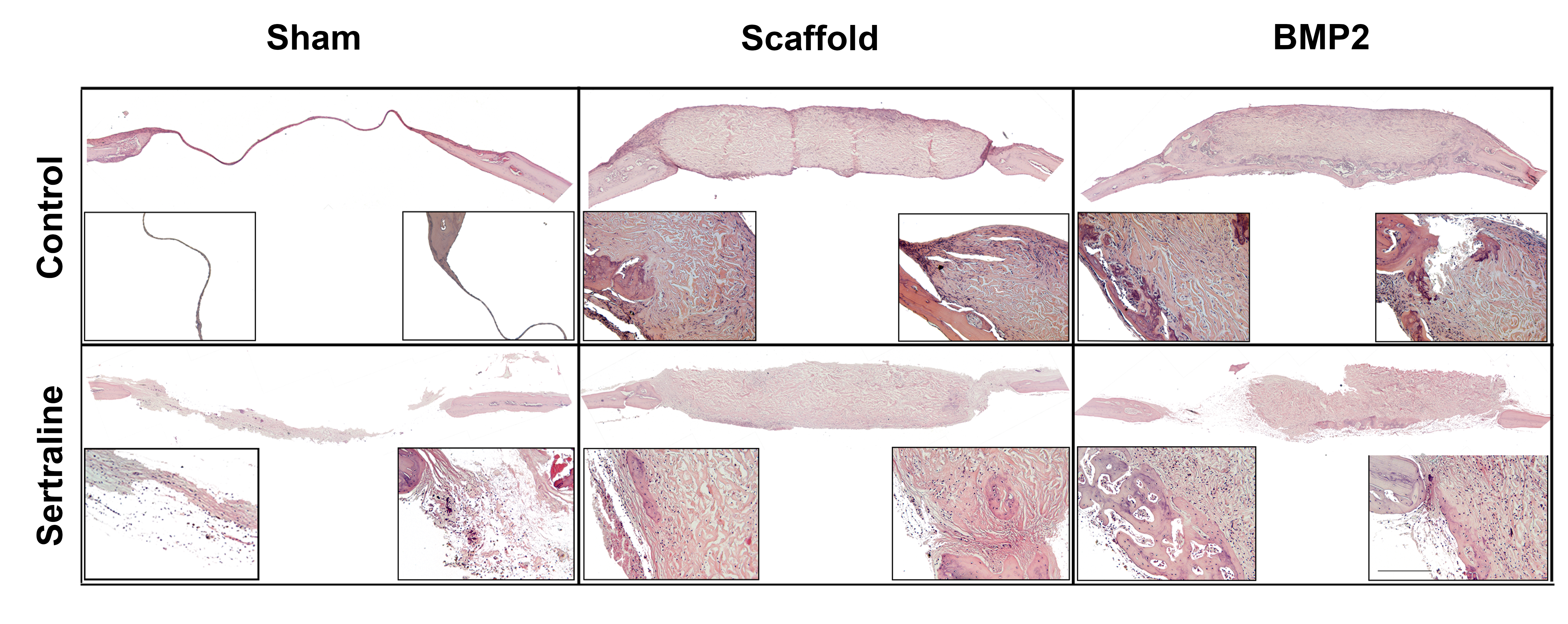

Supplement: Supplementary file 3 — Supplementary Figure 2 [file 41368_2018_26_MOESM3_ESM.tif]

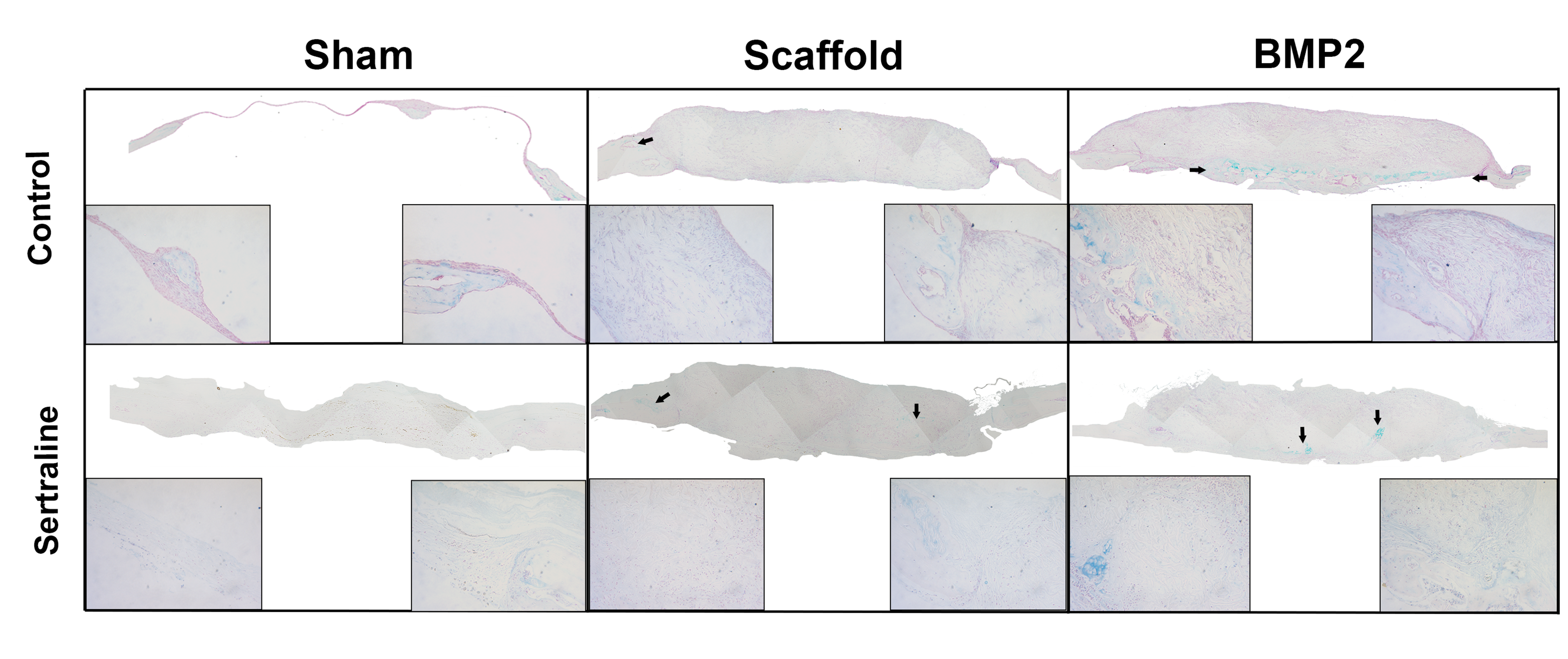

Supplement: Supplementary file 4 — Supplementary Figure 3 [file 41368_2018_26_MOESM4_ESM.tif]

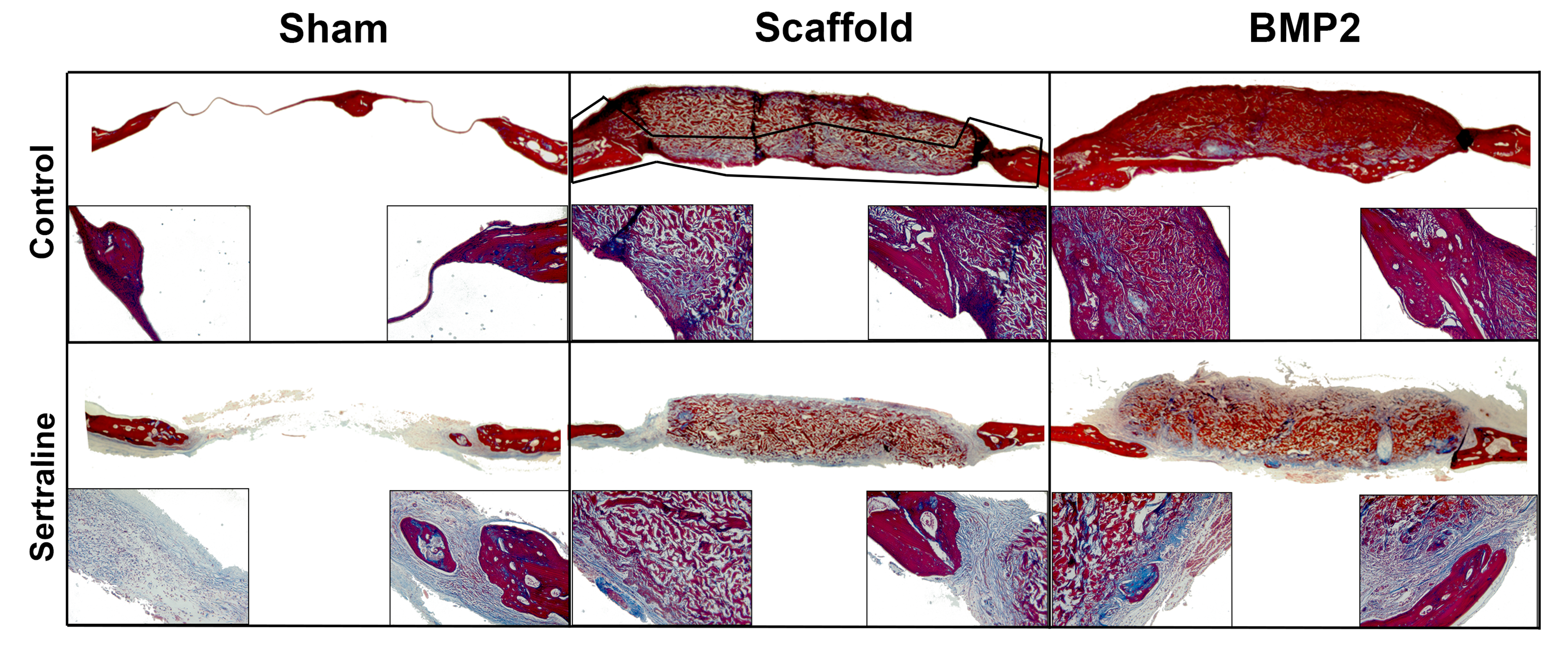

Supplement: Supplementary file 5 — Supplementary Figure 4 [file 41368_2018_26_MOESM5_ESM.tif]

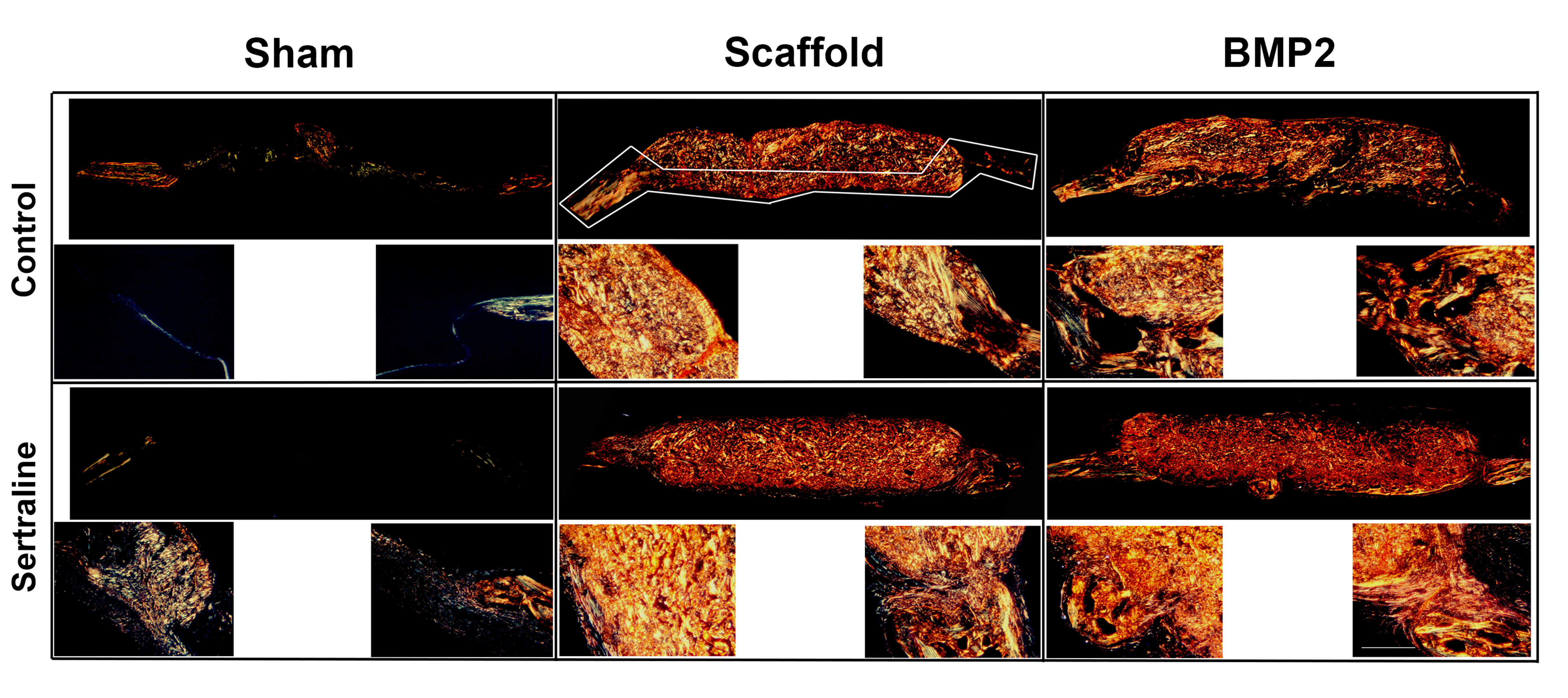

Supplement: Supplementary file 6 — Supplementary Figure 5 [file 41368_2018_26_MOESM6_ESM.tif]

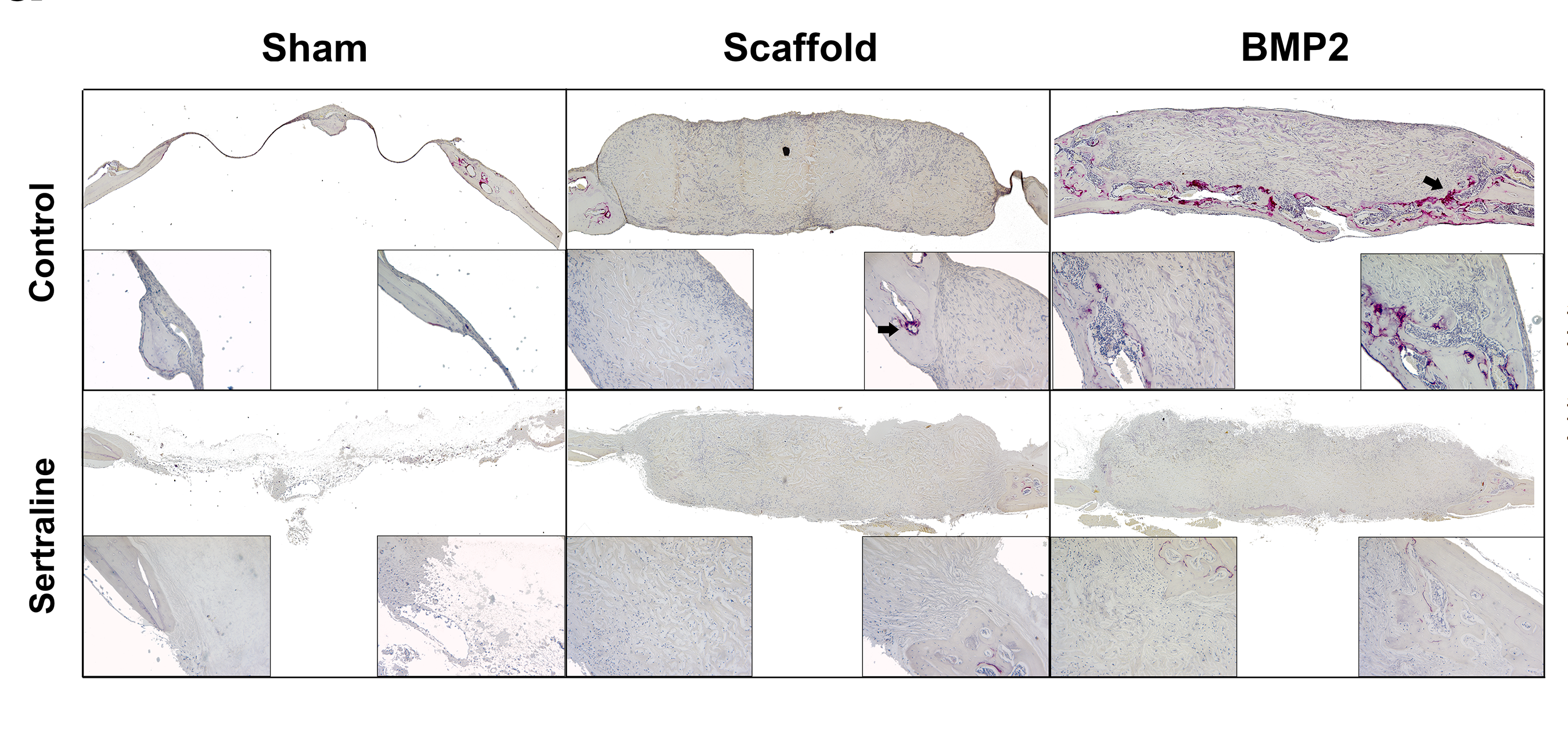

Supplement: Supplementary file 7 — Supplementary Figure 6 [file 41368_2018_26_MOESM7_ESM.tif]

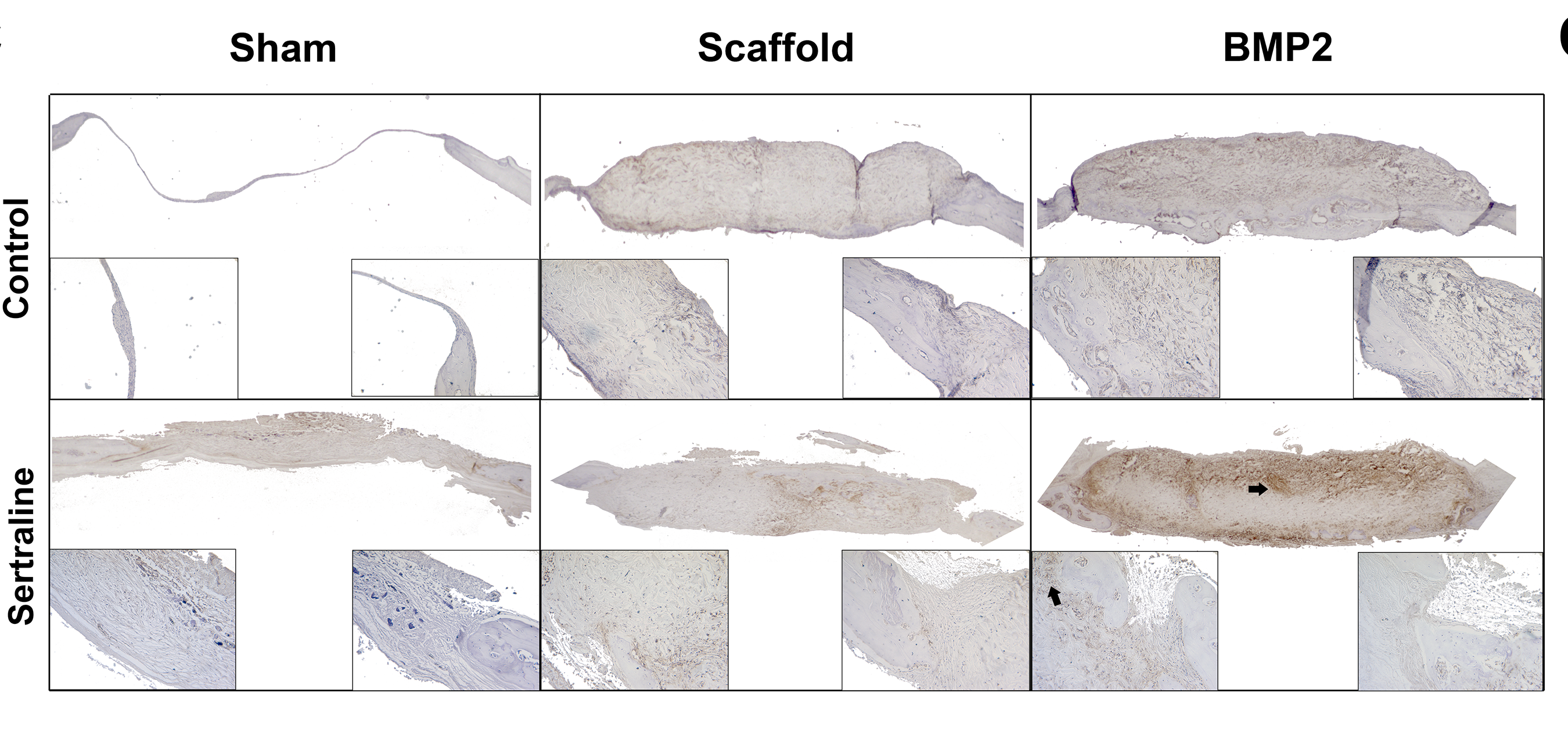

Supplement: Supplementary file 8 — Supplementary Figure 7 [file 41368_2018_26_MOESM8_ESM.tif]

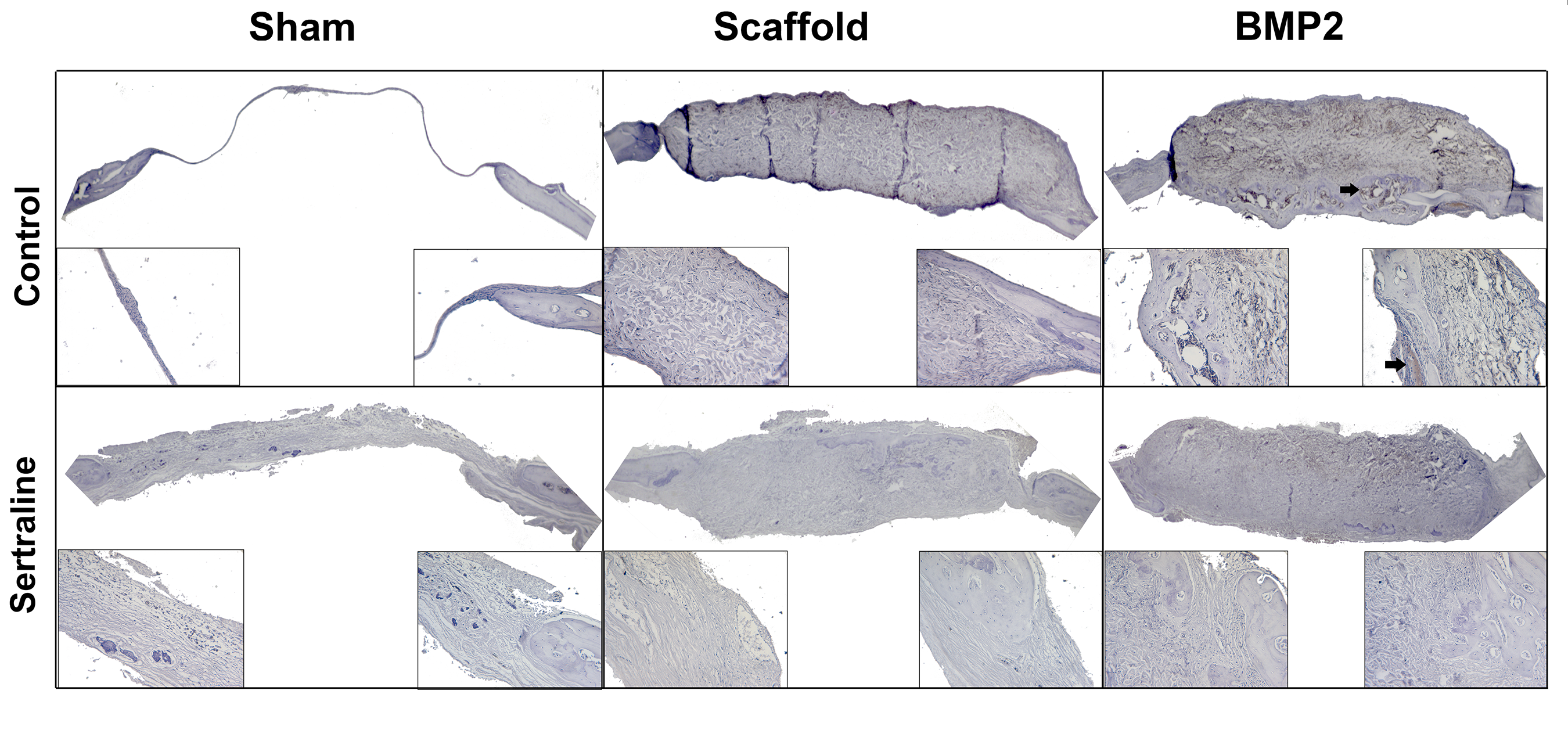

Supplement: Supplementary file 9 — Supplementary Figure 8 [file 41368_2018_26_MOESM9_ESM.tif]

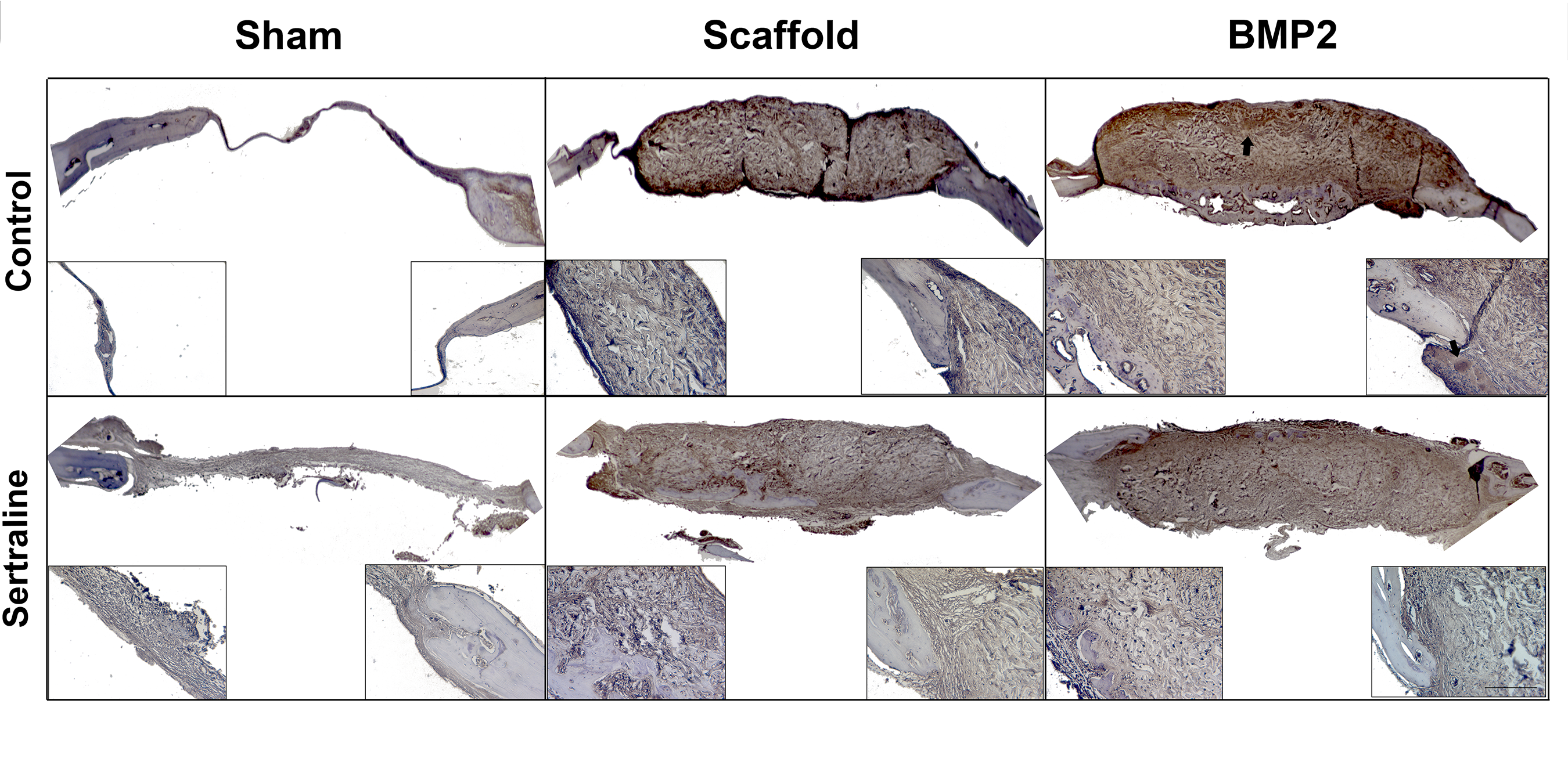

Supplement: Supplementary file 10 — Supplementary Figure 9 [file 41368_2018_26_MOESM10_ESM.tif]

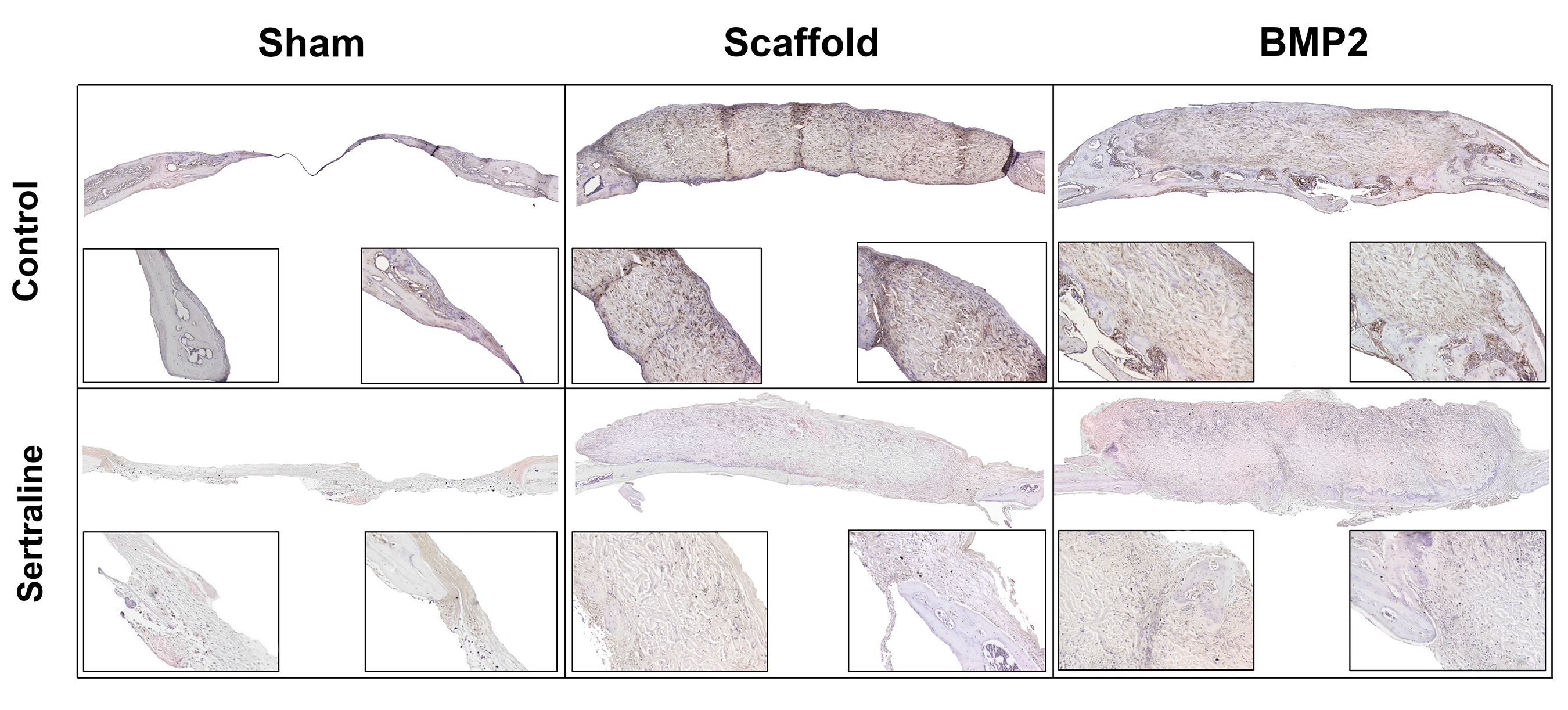

Supplement: Supplementary file 11 — Supplementary Figure 10 [file 41368_2018_26_MOESM11_ESM.tif]

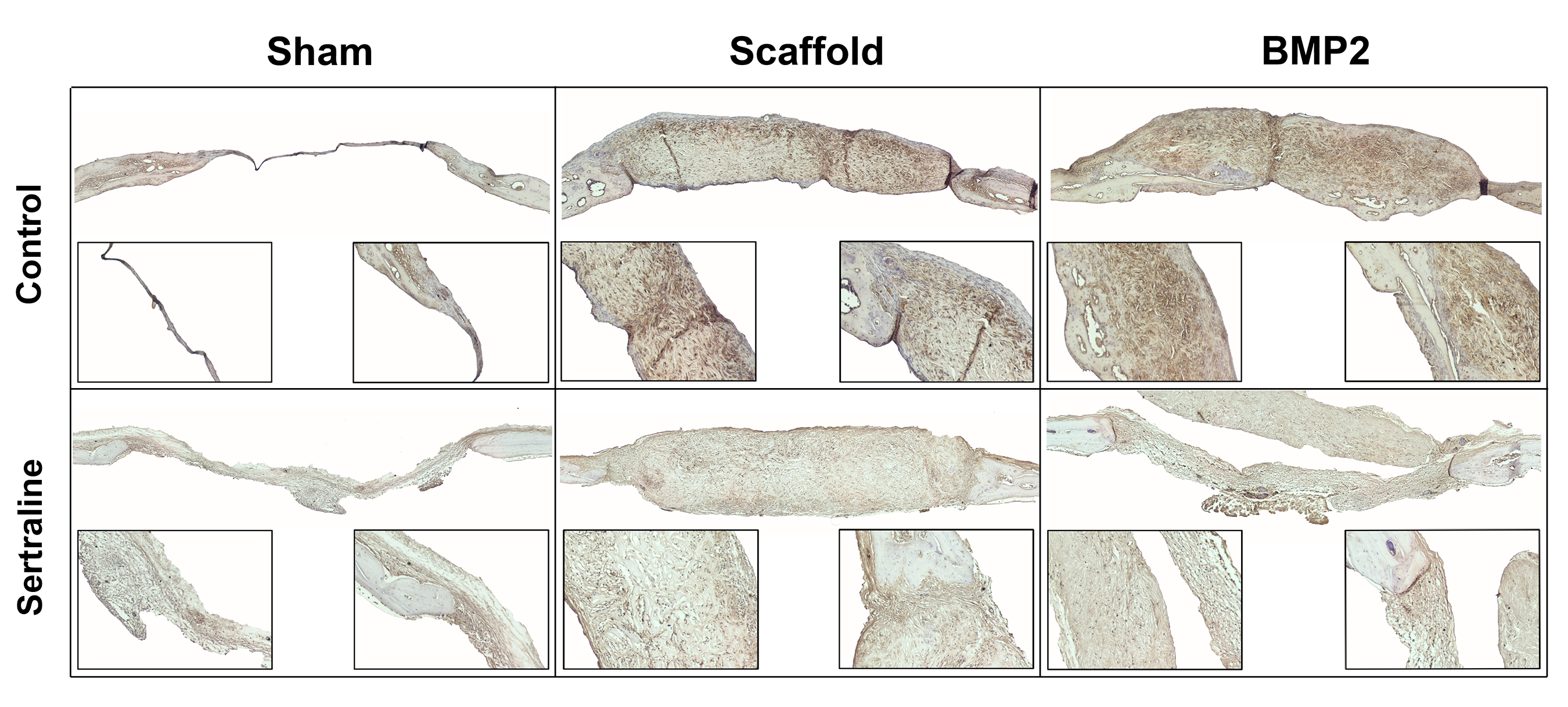

Supplement: Supplementary file 12 — Supplementary Figure 11 [file 41368_2018_26_MOESM12_ESM.tif]
